# Loss of Female Cardiovascular Protection in Diabetes: From Mechanisms to Intervention

**DOI:** 10.3390/cells15211972

**Published:** 2026-10-29

**Authors:** Jillian P. Gady, Shanjida Akter, Mark VandeWiele, Feng Dong

**Affiliations:** Department of Biomedical Sciences, Northeast Ohio Medical University, Rootstown, OH 44272, USA; jgady@neomed.edu (J.P.G.); sakter@neomed.edu (S.A.); mvandewiele@neomed.edu (M.V.)

**Keywords:** diabetes, female cardiovascular protection, sex differences, estrogen, endothelial dysfunction, body composition, physical activity, GLP-1 receptor agonists, SGLT2 inhibitors, cardiovascular risk

## Abstract

**Highlights:**

**What are the main findings?**

- Biological vulnerability (first hit): Diabetes disrupts estrogen-mediated vascular protection, promoting oxidative stress and endothelial dysfunction in women.
- Clinical disadvantage (Second hit): Sex-specific risk factors and clinical care disparities further increase cardiovascular risk in women with diabetes.

**What are the implications of the main findings?**

- A double-hit framework highlights the need for sex-specific cardiovascular risk assessment and prevention strategies for people with diabetes.
- Further research is needed to clarify sex-specific responses to GLP-1 receptor agonists and SGLT2 inhibitors and to optimize cardiovascular prevention in women with diabetes.

**Abstract:**

Premenopausal women without diabetes have a well-established cardiovascular advantage over men of the same age, mainly due to the benefits of estrogen, which has vasoprotective, anti-inflammatory, and antioxidant effects on the vascular endothelium. However, women with type 1 or type 2 diabetes have a 40–50% higher relative risk of coronary heart disease and suffer a greater burden of heart failure and cardiovascular mortality compared with diabetic men. The mechanisms involved in the disparity are not well understood and are seldom considered in clinical guidelines or trials, where women are underrepresented. The paradoxical relationship between chronic hyperglycemia and estrogen signaling plays a critical role, since estradiol, rather than having its usual cardioprotective effect, instead leads to oxidative stress and endothelial dysfunction. This occurs through eNOS uncoupling, activation of the AGE-RAGE axis, and a shift towards pro-inflammatory signaling via estrogen receptors. Sex-related differences in body composition and the distribution of fat tissue also make a significant contribution to the variations in cardiovascular risk. Modifiable lifestyle factors, especially physical activity and skeletal muscle mass, are important targets for intervention, with evidence showing that their benefits on endothelial function, body composition, and systemic inflammation are particularly noticeable in females. In addition, the sex-specific cardiovascular responses to treatments such as glucagon-like peptide-1 receptor agonists (GLP-1RAs) and sodium-glucose cotransporter-2 (SGLT2) inhibitors should be investigated more closely. This review summarizes the current evidence, identifies critical knowledge gaps, and proposes a framework for sex-informed cardiovascular prevention in diabetes.

## 1. Introduction

Cardiovascular diseases remain the leading cause of morbidity and mortality worldwide [1]. Despite this global burden, one consistent observation in cardiovascular epidemiology is that premenopausal women exhibit a cardiovascular advantage, where they are protected against coronary heart disease and stroke relative to age-matched men. In the general population, this female advantage delays the onset of clinically significant cardiovascular disease by approximately a decade, which may contribute meaningfully to the longer life expectancy observed in women across high-income countries. The biological basis of this protection is multifactorial, involving favorable differences in lipid profiles, blood pressure regulation, endothelial function, and systemic inflammation, which are modulated at least in part by the actions of female sex hormones, principally estradiol [2].

However, the onset of diabetes fundamentally disrupts this picture. Since the landmark Framingham Heart Study observations nearly five decades ago, it has been recognized that the usual female cardiovascular protection is erased in the presence of diabetes [3]. Subsequent large-scale meta-analyses have confirmed and quantified this reversal: women with type 2 diabetes bear a 44% greater excess relative risk of coronary heart disease and a 27% greater excess risk of stroke compared with diabetic men [4,5]. The excess burden extends to heart failure, peripheral arterial disease, and cardiovascular mortality. In type 1 diabetes, the disparity is equally pronounced: women face roughly 40% greater all-cause excess mortality and twice the excess risk of fatal and nonfatal vascular events compared with men with the same disease [6]. Critically, these figures represent excess relative risk above the non-diabetic population. While men with diabetes carry a higher absolute burden of disease, women experience a far steeper departure from the cardiovascular safety they would otherwise have. Why diabetes so selectively and completely strips women of their cardiovascular advantage remains an important and underexplored question in cardiometabolic medicine. Biological, hormonal, compositional, behavioral, and clinical factors all appear to contribute, yet they are rarely examined together. This knowledge gap is reinforced by the lack of sex-specific analyses in cardiovascular outcome trials, where women have historically been underrepresented [7]. This long-term underrepresentation has perpetuated a cycle in which treatment guidelines remain sex-neutral despite a sex-specific biological problem.

**Figure 1:**
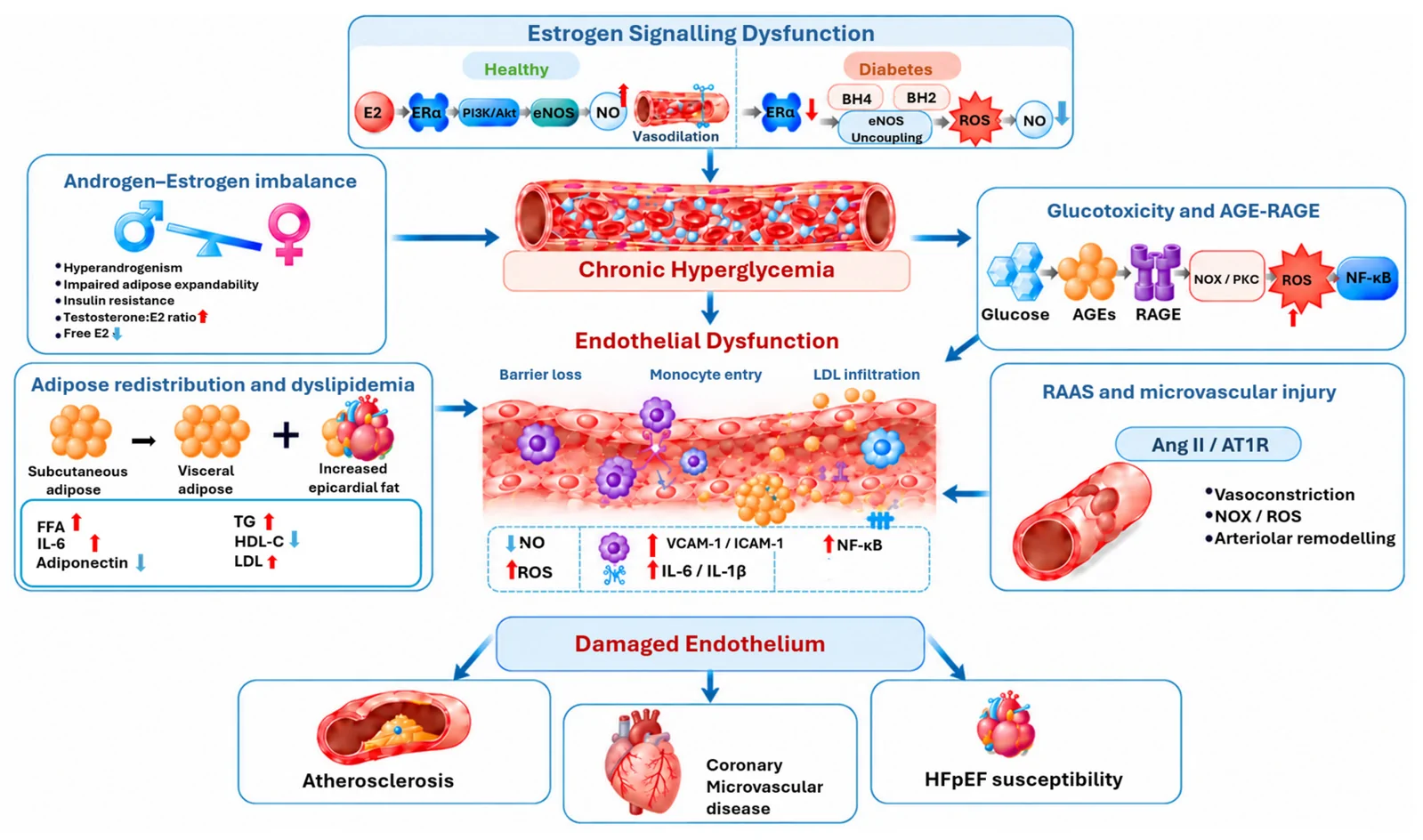
Proposed pathways linking diabetes, estrogen signaling dysfunction, and metabolic disturbances to cardiovascular complications in women. Connecting arrows indicate proposed relationships among the depicted processes and downstream outcomes, whereas upward arrows (↑) and downward arrows (↓) indicate increased and decreased levels, respectively.

**Figure 2:**
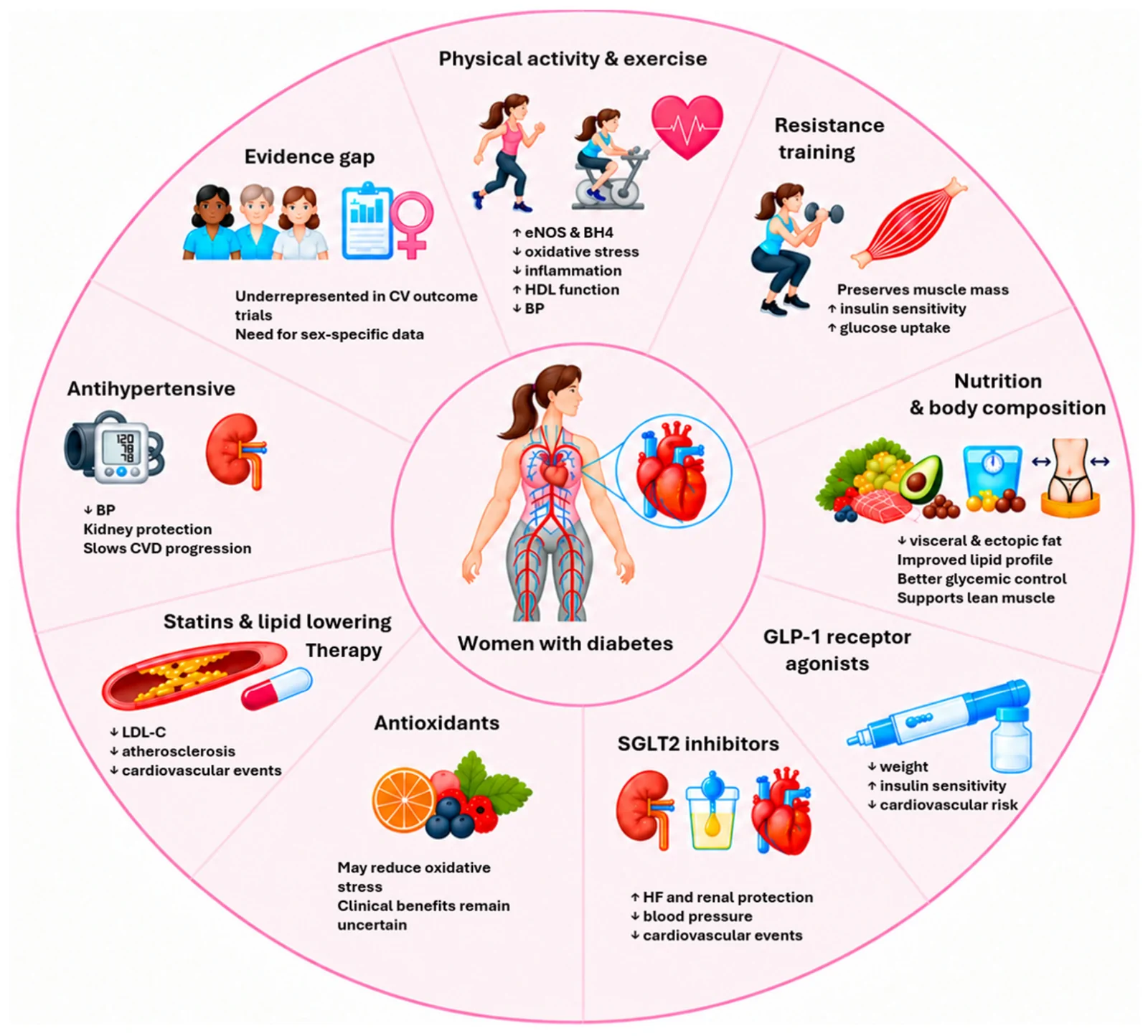
Lifestyle and pharmacological strategies for cardiovascular risk reduction in women with diabetes. Upward (↑) and downward (↓) arrows indicate increased and decreased levels, activity, or risk, respectively.

This review aims to dismantle the sex-neutral approach to diabetic cardiovascular care by examining the unique vulnerability of women through a “double-hit” framework. Hit 1 represents the biological inversion of female cardiovascular protection that is driven by hyperglycemia-induced receptor reprogramming, visceral lipotoxicity, and amplified oxidative stress, summarized in Figure 1. Hit 2 represents the clinical compounding of this biological risk through systemic undertreatment, underrepresentation in clinical trials, and clinical inertia. By mapping both the molecular and clinical dimensions of this disparity, we propose targeted, sex-informed interventional strategies to mitigate female cardiovascular risk (Figure 2).

## 2. The Normal Basis of Female Cardiovascular Protection

### 2.1. Estrogen and Vascular Biology

The cardioprotective effects of estrogen operate through multiple interacting pathways in the vascular endothelium. Estradiol (E2), the principal biologically active estrogen in premenopausal women, exerts both rapid non-genomic effects mediated through membrane-bound estrogen receptors (primarily ERα, ERβ) and G-protein-coupled receptor 30 (GPR30), and slower, sustained genomic effects through nuclear receptor activation [2,8]. Endothelial nitric oxide (NO) bioavailability is the primary molecular mediator in this defense. When bound to its ligand, ERα triggers the PI3K/Akt cascade, phosphorylating and activating endothelial nitric oxide synthase (eNOS), promoting the continuous synthesis of NO [9,10]. NO mediates endothelium-dependent vasodilation, suppresses vascular smooth muscle cell (VSMC) proliferation, and inhibits both platelet aggregation and leukocyte adhesion to the vessel wall. This continuous supply of NO results in a vascular phenotype that is inherently anti-atherogenic.

Beyond stimulating NO generation, estrogen also functions in the protection of NO from degradation. With its antioxidant properties, Estradiol reduces the production of reactive oxygen species (ROS) in endothelial cells, partly by suppressing NADPH oxidase (NOX) activity and limiting the production of superoxide [8,11]. Thus, increased NO production combined with the suppression of ROS production in the endothelium favors vasoprotection. Estrogen also modulates the renin–angiotensin–aldosterone system (RAAS) to favor vasoprotection. The suppression of the RAAS system occurs through the downregulation of the angiotensin II type I receptor (AT_1_R) and the inhibition of angiotensin-converting enzyme (ACE) activity, therefore favoring vasodilation. Estrogen also protects vasculature from systemic inflammation by suppressing NF-κB activation, reducing circulating levels of C-reactive protein (CRP), interleukin-6 (IL-6), and Tumor Necrosis Factor alpha (TNF-α), and limiting monocyte recruitment to the vascular wall [2]. Estrogen receptor activation in cardiomyocytes and vascular smooth muscle cells further contributes to favorable cardiac remodeling and vascular compliance [8]. Collectively, these mechanisms contribute to a robust, multidimensional cardioprotective environment during the reproductively active years.

### 2.2. Metabolic Dimensions of Female Cardiovascular Protection

Female cardiovascular protection is not solely related to endothelial effects, but also an estrogen-driven metabolic phenotype that is part of a defense against lipotoxicity and insulin resistance. Due to estrogen, premenopausal women have a more favorable fat distribution characterized by preferential subcutaneous rather than visceral adiposity. This effectively shields the viscera, liver, and cardiovascular system from the accumulation of ectopic fat [12,13]. This contributes highly to women exhibiting higher HDL cholesterol, lower triglycerides, and lower concentrations of small, atherogenic LDL particles. Simultaneously, women demonstrate greater whole-body insulin sensitivity relative to men of equivalent fitness, driven in part by higher skeletal muscle glucose uptake [14]. These systemic metabolic adaptations combined with the vascular defenses maintain a superior profile of endothelium-dependent vasodilation, leading to lower blood pressure in premenopausal women than age-matched men [10].

The reliance of female physiology on these mechanisms may be demonstrated by menopause. As circulating estradiol levels fall, these advantages begin to converge with and eventually fall below male values. This can be viewed as a natural biological experiment that implicates estrogen as the key driver of their preservation throughout the reproductive years [2]. Consequently, the premenopausal metabolic profile is not innate, but actively maintained by an estrogen-dependent state that is pathologically disrupted under diabetic conditions.

## 3. Hit 1: Biological Mechanisms Erasing Female Cardiovascular Protection

### 3.1. The Estrogen Paradox in Diabetes

One of the most compelling mechanistic hypotheses rising in this field is that estrogen, normally cardioprotective, may become maladaptive in the presence of diabetes. Studies in premenopausal women with type 1 diabetes suggest that endothelial function, measured by flow-mediated dilation (FMD), actually decreases from the menstrual phase to the late follicular phase (when estradiol concentrations are highest), in direct contrast to the expected FMD increase observed in healthy women across the same hormonal transition [15]. This inversion of the normal estrogen–endothelial relationship represents a fundamental disruption of the female cardiovascular advantage. The underlying mechanism centers on the interaction between chronic hyperglycemia and estrogen receptor signaling. Under physiological conditions, ERα is the primary receptor mediating cardioprotection, coupling to the PI3K/Akt pathway to stimulate NO production [16,17]. In contrast, chronic hyperglycemia and sustained oxidative stress downregulate ERα expression while maladaptively preserving or upregulating ERβ and GPR30 in the diabetic endothelium. As a consequence, E2 signaling is shunted away from its normal PI3K/Akt axis, creating a receptor imbalance. With the ERα-PI3K/Akt pathway compromised, estradiol signaling is actively directed toward these altered receptor populations amidst severe oxidative stress. Concurrently, oxidation of the eNOS cofactor tetrahydrobiopterin (BH4) into the inactive radical BH2 uncouples the eNOS enzyme [18,19]. Consequently, the follicular-phase estradiol surge acts upon this unbalanced receptor profile and uncoupled eNOS system to actively amplify superoxide generation, converting a physiological vasodilatory stimulus into a mechanism of vascular injury [15,20]. This mechanistic inversion may help explain why the female hormonal environment that confers protection in health becomes a driver of harm in diabetes.

It is important to explicitly note the evidentiary weight of this narrative. Clinical evidence for this in vivo inversion is currently sparse, coming primarily from premenopausal women with type 1 diabetes [15]. Whether this paradox operates identically in type 2 diabetes remains to be clinically validated. However, the compounding effects of insulin resistance, visceral adiposity and systemic inflammation inherent to type 2 diabetes establish an oxidative environment that is likely capable of driving parallel receptor disruption. Furthermore, because this paradox relies on cyclic surges of endogenous estradiol acting upon damaged endothelium, the cyclic amplification of oxidative stress is strictly a premenopausal and perimenopausal phenomenon. Following menopause, this cyclic oxidative surging subsides as estradiol levels decline, yet the underlying receptor downregulation and eNOS uncoupling leave the vasculature chronically compromised.

### 3.2. Androgens, Cardiovascular Health, and Diabetes

The role of androgens and their effect on female cardiovascular health is inconclusive. While some studies argue that increased levels of androgens are associated with CVD in females, others have argued that no such association exists [21,22]. While androgens may not be conclusively linked to CVD, they can be associated with many factors associated with diabetes. Research has found that hyperandrogenism in women has been linked to specific obesity factors, inhibition of adipose hyperplasia, and insulin resistance, indicating a possible link between hyperandrogenism and diabetes [23]. Finally, diabetes and cardiovascular health have been linked to imbalance of androgen/estrogen ratios in women. In a 2023 study, it was found that women with type 1 diabetes and cardiovascular autonomic dysfunction had higher total testosterone/E2 and free testosterone/E2 molar ratios, lower E2, and free E2, compared with those without cardiovascular autonomic dysfunction [24]. These results suggest that increased levels of androgens in females with diabetes could impact cardiovascular health. However, further research is needed to confirm the link between androgens, diabetes, and cardiovascular health in women.

### 3.3. The AGE-RAGE Axis and Sex-Specific Endothelial Vulnerability

Advanced glycation end-products (AGEs), the irreversible products of non-enzymatic glycation that accumulate in proportion to cumulative glycemic exposure, are central mediators of diabetic vascular injury. Binding AGEs to their receptor for AGEs (RAGE) activates NF-κB, increases NADPH oxidase activity, stimulates the production of proinflammatory cytokines, and directly suppresses eNOS expression and phosphorylation [19,25]. This combination of reduced nitric oxide (NO) bioavailability and increased oxidative stress contributes to endothelial dysfunction, accelerates atherogenesis, and increases vascular stiffness. Emerging preclinical and clinical data suggest that female endothelial cells may be more sensitive to hyperglycemia-induced mitochondrial dysfunction than male cells, exhibiting more pronounced impairments in vasodilation, angiogenesis, and mitochondrial activity under diabetic conditions [26]. In a healthy state, estrogen maintains the endothelial mitochondrial networks through the upregulation of peroxisome proliferator-activated receptor-gamma coactivator-1 alpha (PGC-1α), which is the key regulator of mitochondrial biogenesis and antioxidant defense [27]. In the diabetic environment, ERα downregulation and intracellular glucose toxicity suppress PGC-1α expression, leaving female endothelial cells unable to clear damaged mitochondria or generate healthy replacements as effectively. This leads to electron leak and ROS generation that can overwhelm the antioxidant defenses [28]. Although these findings remain incompletely understood and require more in-depth scientific study, they suggest that the female endothelium may depend more heavily on estrogen-mediated mitochondrial homeostasis that, when disrupted by the diabetic environment, leaves female cells disproportionately exposed to oxidative insult.

### 3.4. Oxidative Stress and Inflammation: A Female-Amplified Loop

Chronic hyperglycemia generates oxidative stress through multiple converging pathways: excess glucose flux through the polyol pathway, increased protein kinase C (PKC) activation, mitochondrial electron transport chain overload, and AGE-RAGE-mediated NOX activation [19]. In women with diabetes, these pathways intersect with the disrupted estrogen receptor signaling described above to create an amplified oxidative and inflammatory state [29]. A key mechanism underlying this amplification is the loss of estrogen-mediated transrepression of inflammatory signaling. Under physiological conditions, estrogen receptors with their ligands exert anti-inflammatory effects through the upregulation of IκBα (an NF-κB inhibitor) and binding to the p65 subunit of the NF-κB complex. This interaction blocks p65 from binding to the DNA promoter of inflammatory genes [30]. In the hyperglycemic state of diabetes, AGE accumulation and ROS production, this inhibitory crosstalk is dismantled. IκBα is heavily degraded, and with suppressed estrogen signaling, it is not replaced, resulting in sustained NF-κB activation. Consequently, NF-κB promotes the expression of key inflammatory mediators, including vascular cell adhesion molecule-1 (VCAM-1), intercellular adhesion molecule-1 (ICAM-1), and monocyte chemoattractant protein-1 (MCP-1), thereby exacerbating endothelial inflammation and vascular dysfunction [19].

Systemic markers of inflammation including high-sensitivity CRP, IL-6, and fibrinogen are disproportionately elevated in women with diabetes compared with diabetic men, and the relationship between inflammatory burden and cardiovascular outcomes appears steeper in women [29]. Ultimately, the diabetic environment appears to convert what is normally an anti-inflammatory signaling axis into a driver of vascular NF-κB activation, enhanced expression of inflammatory mediators, and monocyte adhesion.

Although men with diabetes also experience AGE-RAGE-induced NF-κB activation, their baseline vascular homeostasis is less dependent on estrogen-mediated transrepression. In contrast, women with diabetes experience both the direct pro-inflammatory effects of chronic hyperglycemia and a collapse of their primary endothelial defense mechanism mediated by ER signaling, which may contribute to the loss of the IκBα “shield” and a steeper escalation of inflammatory markers and a sharper increase in cardiovascular risk.

### 3.5. Dyslipidemia: A Greater Deterioration in Women

The transition from normoglycemia to overt diabetes is associated with a more profound and destructive deterioration of the lipid profile in women than in men [4,12]. Because premenopausal women preferentially utilize subcutaneous fat storage for excess lipids, women typically develop clinically apparent diabetes at a higher BMI than men, and fat eventually is redirected to visceral depots. This metabolic transition results in greater diabetes-associated reductions in HDL cholesterol, greater increases in triglycerides, and a more pronounced shift toward small dense LDL particles—changes that track more strongly with cardiovascular events in women than in age-matched men [12,31]. The mechanisms are related to the interaction between impaired insulin signaling, adipose tissue dysfunction, and hepatic lipoprotein metabolism; the preferential subcutaneous fat distribution of premenopausal women appears to mask metabolic deterioration during early adiposity expansion, but when visceral and ectopic fat eventually accumulate in diabetes, the resulting dyslipidemia is more and atherogenic per unit of adiposity than in men [12,13].

### 3.6. Blood Pressure, RAAS, and Microvascular Injury

Hypertension is more strongly associated with cardiovascular outcomes in women with diabetes than in men [29], making it a sex-specific multiplier driving their cardiovascular morbidity and mortality. In healthy vasculature, estrogen normally modulates RAAS to favor angiotensin-(1–7) and bradykinin-mediated vasodilation and suppresses vasoconstrictive AT_1_R signaling [8]. In the diabetic environment, this hormonal modulation is disrupted. The resulting RAAS imbalance drives endothelial inflammation, sustained oxidative stress, and rapid arteriolar remodeling [8,19]. The female microvasculature is more dependent on estrogen-mediated vasodilatory tone; therefore, the unchecked RAAS activation leaves female arteriolar beds more vulnerable to injury. This susceptibility helps explain why women with diabetes are disproportionately affected by microvascular disease, including ischemic heart disease with non-obstructive coronary arteries (INOCA), a particularly dangerous phenotype that does not match the traditional macrovascular risk that consistently treats male physiology as the baseline, and therefore presents an important clinical blind spot [13,32].

Table 1 summarizes the mechanisms underlying the loss of female cardiovascular protection in women with diabetes.

The mechanisms described above are closely linked to the changes in adipose tissue structure and metabolism. In women with diabetes, increased visceral and ectopic fat is not only a consequence of metabolic dysfunction but also a contributor to endothelial dysfunction, oxidative stress, and inflammation. For example, visceral adipose tissue releases free fatty acids and pro-inflammatory adipokines into the portal circulation, which can enhance AGE-RAGE–mediated NF-κB activation and further impair estrogen receptor signaling that normally helps limit vascular inflammation. Additionally, epicardial fat may also have local lipotoxic and inflammatory effects on the coronary endothelium, adding to the eNOS uncoupling and mitochondrial dysfunction caused by chronic hyperglycemia. Thus, when subcutaneous adipose tissue can no longer adequately store excess energy, the resulting accumulation of visceral and ectopic fat does more than add another cardiovascular risk factor. It can further amplify the molecular pathways that contribute to loss of the female vascular protective effect. For this reason, changes in body composition in women with diabetes should be considered an important part of the mechanisms underlying their increased cardiovascular risk (Figure 1).

## 4. Body Composition, Fat Distribution, and Sex-Specific Cardiovascular Risk

### 4.1. Beyond BMI: Why Body Composition Matters Differently in Women

BMI is a notoriously imperfect measure of cardiometabolic risk, and its limitations are particularly pronounced in women. This is highly relevant in the pathogenesis of type 2 diabetes, where body composition heavily dictates metabolic changes. Women generally carry a higher percentage of total body fat than men at the same BMI, concentrated preferentially in subcutaneous depots, a pattern that is metabolically relatively benign compared with visceral adiposity [13,32]. Because of the large storage capacity of subcutaneous fat, women typically develop diabetes at higher BMI values than men, having accumulated more total adipose tissue before reaching the visceral fat thresholds associated with insulin resistance, dyslipidemia, and inflammation [4,12]. This dynamic obscures early cardiometabolic risk: by the time clinical diabetes is diagnosed, women have often already undergone a substantial metabolic change in the redistribution of fat toward visceral and ectopic depots after exhausting subcutaneous storage. As a consequence, the severity of metabolic tissue disruption at the time of diagnosis may be greater in women than in men despite similar or lower BMI [12], as well as the resulting cardiovascular threat.

### 4.2. Visceral and Ectopic Fat Depots

Once fat begins accumulating viscerally as visceral adipose tissue (VAT) and ectopic depots, a distinct and more destructive secretory profile is generated. Compared with subcutaneous fat, VAT releases higher concentrations of pro-inflammatory adipokines (including leptin, resistin, and IL-6), free fatty acids, and ROS into the portal circulation. This directly exposes the liver and cardiovascular system to lipotoxic and inflammatory signals [13,32]. In women with diabetes, accumulation of VAT is associated with disproportionate reductions in adiponectin, which is a cardioprotective adipokine that normally promotes endothelial NO production, reduces vascular inflammation, and improves insulin sensitivity [13]. The accumulation of VAT in women is also associated with amplified systemic inflammatory responses compared with similarly obese men [13,32].

Ectopic fat deposition is the accumulation of fat in pericardial, hepatic, intramyocardial, and skeletal muscle compartments, which represents an additional and underappreciated dimension of sex-specific cardiovascular risk in diabetes. This can be considered even more dangerous than VAT, particularly epicardial adipose tissue (EAT). EAT is in direct anatomical contact with the coronary arteries and myocardium, lacking separation by fascia. As EAT expands in diabetic women, it exerts toxicity on the heart through a paracrine mechanism, which is associated with coronary microvascular dysfunction, myocardial steatosis, and adverse left ventricular stiffening. In turn, this contributes to the disproportionate burden of heart failure with preserved ejection fraction (HFpEF) observed in women with diabetes and obesity [13,29].

### 4.3. Skeletal Muscle Mass as a Cardioprotective Factor

Skeletal muscle is the primary site of insulin-stimulated glucose disposal. Consequently, muscle mass is a critical determinant of whole-body insulin sensitivity and metabolic health [14]. In women with diabetes, low skeletal muscle mass (sarcopenia or relative muscle depletion) is common, and when compounded with the effects of aging, insulin resistance and obesity, a phenotype that is increasingly recognized as “sarcopenic obesity” Occurs. This combination of low muscle mass and high visceral adiposity in women with diabetes is associated with higher cardiovascular risk. Without skeletal muscle to uptake glucose, these women suffer from elevated circulating free fatty acids and reduced cardiorespiratory fitness that is independent of total body fat [14,33]. Physical fitness, a functional index of skeletal muscle and cardiorespiratory capacity, is among the strongest predictors of cardiovascular mortality in both T1D and T2D, with cardiorespiratory fitness showing an inverse dose–response relationship with cardiovascular events [34,35]. Maintaining muscle mass and aerobic fitness therefore represents not only a general health goal, but also an absolute cardiovascular imperative for women with diabetes.

## 5. Hit 2: Clinical Disparities and Behavioral Compounding Factors

### 5.1. Disparities in Cardiovascular Risk Factor Management

The excess biological risk experienced by women with diabetes is further compounded by systematic disparities in the clinical management of cardiovascular risk factors. Multiple studies consistently suggested that women with diabetes are significantly less likely to be prescribed lipid-lowering therapies and RAAS inhibitors, less likely to achieve recommended LDL cholesterol or blood pressure targets, and less likely to receive antiplatelet agents after cardiovascular events compared with diabetic men [31,36,37,38]. These disparities persist even after adjustment for relevant clinical variables and appear to reflect both provider-level underestimation of cardiovascular risk in women and patient-level undertreatment [36,37]. Notably, one study of over 10,000 individuals with type 2 diabetes found that women were significantly less likely to be prescribed statins and RAAS inhibitors, and these disparities were independently associated with their higher excess risks for cardiovascular and renal complications [38]. These disparities solidify the “second hit” of the female cardiovascular phenotype, as medical providers underestimate diabetic female cardiovascular risk and their previously discussed biological vulnerability. The combination of biological vulnerability and medical undertreatment can lead to adverse outcomes if left unaddressed.

### 5.2. Underrepresentation in Cardiovascular Outcome Trials

The medical undertreatment of women has roots in their historical exclusion from cardiovascular outcome trials (CVOTs). Women have historically comprised only 28–36% of participants in CVOTs of diabetes medications [7]. This underrepresentation constitutes a fundamental, longstanding limitation in cardiovascular research. It constrains the detection of sex-specific differences in treatment efficacy, safety, and mechanism. Trials that are predominantly male limit the identification of female subgroups that may experience disproportionate benefit or harm, and weaken the sex-stratified evidence required to inform clinical guidelines [7,29]. The consistent sex neutrality of current cardiovascular risk guidelines for diabetes reflects, in part, the sex-neutral composition of the trials on which they rest, a circularity that perpetuates undertreatment of women.

### 5.3. Female-Specific Life Events as Additional Risk Layers

Beyond traditional cardiovascular risk factors, several female-specific reproductive and endocrine events further influence cardiovascular risk in women with diabetes. Pregnancy involves profound changes to the cardiovascular system and metabolism. These changes include, but are not limited to, increased insulin resistance, hypercoagulability, and elevated lipid levels, which may have long-lasting health impacts [39]. A 2019 systematic review and meta-analysis found that a history of adverse pregnancy outcomes (APO), specifically preeclampsia, gestational hypertension, gestational diabetes mellitus, placental abruption, preterm birth, and stillbirth elevates a woman’s risk of subsequent CVD and cerebrovascular morbidity and mortality [40]. Epidemiological evidence reveals that hypertensive APOs, such as preeclampsia, may cause persistent vascular changes, and statistics have suggested that women with T1D have a higher risk of preeclampsia compared to those without the condition (10–17% vs. 3–5%) [39]. This compounding of cardiovascular risk factors is particularly concerning, as studies link preeclampsia to subclinical atherosclerosis [41].

Gestational diabetes (GDM) is now recognized as a powerful sex-specific cardiovascular risk and prognostic marker: a recent meta-analysis involving over 5.3 million women suggested a twofold higher risk of CVD in women with previous GDM, emerging within the first decade post-pregnancy, and this risk remained elevated even when women who subsequently developed type 2 diabetes were excluded [42]. The combination of GDM history and subsequent type 2 diabetes development accelerates atherosclerosis progression and coronary microvascular dysfunction.

Furthermore, an increasingly recognized risk layer is the condition traditionally known in cardiovascular literature as Polycystic Ovary Syndrome (PCOS). Although readers may be encountering the updated term for the first time here, this review utilizes the recently proposed nomenclature that is Polyendocrine Metabolic Ovarian Syndrome (PMOS), to better represent its widespread endocrine, metabolic and systemic nature. This is a disorder that affects 5–15% of women of reproductive age and confers an independently elevated cardiovascular risk [43]. PMOS intersects additively with diabetic cardiovascular pathology, as it is strongly associated with increased insulin resistance and type 2 diabetes prior to the onset of menopause [44]. Finally, menopause represents a critical acceleration point: the abrupt decline in circulating estradiol triggers rapid deterioration of endothelial function, accelerated visceral fat redistribution, worsening dyslipidemia, and rising blood pressure. These changes in women with pre-existing diabetes compound an already adversely remodeled cardiovascular risk profile [2,45].

### 5.4. Psychosocial and Behavioral Factors

Navigating a chronic metabolic disease involves a substantial psychosocial burden that is often underestimated in those without firsthand experience. This burden can act as an independent cardiovascular risk multiplier. Depression and diabetes distress are significantly more prevalent in female patients, and both are independently associated with adverse cardiovascular outcomes. These outcomes are not driven solely through behavioral disengagement, but are mediated through direct physiological pathways that include heightened hypothalamic–pituitary–adrenal (HPA) axis activation, sympathetic nervous system dysregulation, poor medication adherence, and reduced engagement with cardiovascular-protective behaviors including physical activity [29]. Disordered eating and insulin omission, a behavior used to induce weight loss through glycosuria, are female-predominant phenomena in patients with type 1 diabetes, particularly in adolescence and early adulthood, and are associated with chronic hyperglycemic exposure, accelerated complications, and elevated cardiovascular mortality [46]. For both type 1 and type 2 diabetic patients, restrictive dieting behaviors, emotional eating, and weight cycling are practices more prevalent in women, which may impede their adherence to cardioprotective dietary and nutritional guidelines, potentially leading to exacerbated glycemic fluctuation and metabolic dysregulation [47].

## 6. Intervention: Restoring or Preserving Female Cardiovascular Protection

Lifestyle modifications and pharmacological interventions are critical for restoring cardiovascular protection in women. Regular physical activity significantly improves vascular and metabolic health in individuals with diabetes [34]. Pharmacological therapies, including GLP-1 receptor agonists, SGLT2 inhibitors, lipid-lowering agents, and antihypertensive medications, have demonstrated efficacy in reducing cardiovascular risk (Figure 2) [48,49]. Table 2 summarizes clinical evidence supporting cardiovascular risk-reduction interventions in type 1 and type 2 diabetes and highlights gaps in evidence related to women identified in clinical trials.

### 6.1. Physical Activity and Exercise

Regular physical activity is among the most potent and broadly accessible interventions for reducing cardiovascular risk in women with diabetes, operating through mechanisms that directly address the key drivers of lost female protection. Therefore, regular physical exercise should be increasingly regarded as an effective targeted medical intervention, rather than general lifestyle advice. Aerobic exercise actively restores endothelial NO bioavailability by upregulating eNOS expression and restoring BH4 levels, effectively reversing the eNOS uncoupling that underlies the previously discussed estrogen paradox in diabetes [34,53]. It reduces circulating markers of oxidative stress and systemic inflammation, improves HDL functionality, lowers blood pressure, and favorably shifts fat distribution away from visceral and ectopic depots [34,35]. In type 1 diabetes specifically, high-intensity interval training (HIIT) produces superior improvements in endothelial function and cardiorespiratory fitness compared with moderate-intensity continuous training, with FMD improvements demonstrable after 8 weeks [53]. In both T1D and T2D, aerobic training rapidly increases cardiorespiratory fitness, reduces HbA1c, improves lipid profiles, and measurably improves endothelial function [34].

Resistance training can act as a necessary complement to aerobic exercise by preserving and building skeletal muscle mass, a “metabolic sink” in the human body. Skeletal muscle is the body’s primary disposal site for circulating energy. Skeletal muscle absorbs the majority of post-meal glucose, burns fatty acids for resting energy, and protects organs from metabolic overload. Glucose uptake by skeletal muscle can occur independent of insulin, allowing the muscles to clear blood sugar, even in a state of insulin resistance [58,59]. Resistance training, which focuses on weight training to build muscle, can improve insulin-stimulated glucose disposal, reduce free fatty acid flux, and support cardiorespiratory fitness indirectly through improvements in lean body mass [33,35]. A 2025 systematic review and meta-analysis of 25 randomized controlled trials in T1D confirmed that exercise training reduces BMI, fasting glucose, and HbA1c and increases Maximal oxygen uptake (VO_2_max), with age, exercise mode, and intervention duration as key moderators of effect [35]. Given the particular importance of skeletal muscle mass for cardiovascular function in women, a combined aerobic and resistance training approach offers synergistic benefits.

Despite the clear evidence for these benefits, physical activity levels are substantially lower in women with diabetes than in men, and exercise recommendations have historically been based on predominantly male populations [73]. Societal pressure, widespread and constantly changing beauty standards, and social media often lead women to desire thinness, even at the expense of their health [74]. Sex-stratified evidence on optimal exercise modality, intensity, and duration for cardiovascular protection in women with diabetes remains limited, particularly for type 1 diabetes, representing an urgent research gap [73]. Educating the public on the importance of exercise and the evidence-based differences it can make on cardiovascular health and longevity remains critical, especially as societal pressures shift ideal body types in and out of trends.

### 6.2. Body Composition-Targeted Interventions

Therapeutic paradigms must shift and change focus from total body weight to body composition remodeling. Targeted reduction in visceral and ectopic fat represents a more precise cardiovascular intervention goal than weight loss per se [13,32]. With VAT contributing to female-specific risk, lifestyle interventions that reduce VAT are important to relieve circulation from lipotoxic stress. Combining caloric modification with physical activity consistently reduce VAT and improve cardiovascular risk markers, and these benefits are disproportionate relative to total weight lost, reflecting the metabolic sensitivity of visceral fat to caloric restriction and physical activity [13]. The preservation of skeletal muscle mass during weight loss is a critical and often overlooked objective in women with diabetes. Conventional caloric restriction without concurrent resistance exercise results in significant lean mass loss alongside fat loss, potentially undermining the insulin-sensitizing and cardiovascular benefits of the intervention [33,35]. Resistance training during intentional weight loss programs effectively preserves lean mass, and the combination of adequate dietary protein intake with resistance exercise represents the most evidence-based approach to favorable body composition remodeling in women with diabetes.

### 6.3. Pharmacological Interventions with Sex-Specific Potential

#### 6.3.1. GLP-1 Receptor Agonists

GLP-1 receptor agonists (GLP-1 RAs) have emerged as major therapeutic advances in the management of cardiovascular risk in type 2 diabetes, and their clinical utilization has soared in recent years. Landmark trials suggest significant reductions in major adverse cardiovascular events with the use of these medications [49,75]. The SOUL trial (2025) demonstrated that oral semaglutide may reduce major adverse cardiovascular events (MACE) by 14% (HR 0.86; 95% CI, 0.77–0.96; *p* = 0.006) in patients with T2D and established atherosclerotic cardiovascular disease or chronic kidney disease, over a median follow-up of approximately 49.5 months, primarily driven by reductions in non-fatal myocardial infarction [75,76]. Beyond glycemic control, GLP-1 RAs exert anti-inflammatory and anti-atherogenic effects on the vascular endothelium, reduce visceral fat mass and total body weight, lower blood pressure, and improve endothelial function [49]. The preferential reduction in visceral adiposity associated with GLP-1 RA therapy is particularly relevant for women with diabetes, given the centrality of visceral fat expansion to the loss of female cardiovascular protection [13,32]. Women are disproportionately prescribed these therapies for both glycemic control and weight management; however, they remain systematically underrepresented in CVOTs that define their efficacy. Sex-stratified analyses from large GLP-1 RA trials remain incompletely reported, representing a pressing evidence gap [7]. While the mechanism aligns favorably with female-specific visceral fat patterns, the lack of sex-stratified outcome data prevents definitive conclusions regarding superior efficacy in women.

#### 6.3.2. SGLT2 Inhibitors

SGLT2 inhibitors are known to reduce cardiovascular mortality and heart failure hospitalizations in patients with type 2 diabetes and established cardiovascular disease, with benefits extending to patients without diabetes in the setting of heart failure [49,75]. Their cardiovascular effects operate through multiple non-glycemic mechanisms including natriuresis-mediated blood pressure reduction, hematocrit elevation, reduction in cardiac preload and afterload, and potentially direct cardiomyocyte effects on mitochondrial function and energetics [49]. Sex-stratified data from major SGLT2 inhibitor trials are inconsistent, with some analyses suggesting attenuated benefits in women, an observation that requires mechanistic investigation [7]. As new pharmacological interventions continue to be developed, it is important to also improve knowledge on medications that evidence has already proven to be effective, to maximize their benefit in all populations.

#### 6.3.3. Statins and Lipid-Lowering Therapy

Statins reduce cardiovascular events in women with diabetes at least as effectively as in men on a relative basis, yet women with diabetes remain systematically undertreated with lipid-lowering therapy and are less likely to reach LDL targets [31,37,38]. Women remain under-screened and undertreated for dyslipidemia compared with men, and also comprise a small proportion of clinical trial participants for lipid-lowering agents [31]. Given that dyslipidemia, particularly the HDL reduction and triglyceride elevation associated with diabetic metabolic dysregulation, is a particularly strong cardiovascular risk driver in women, aggressive lipid management is arguably more urgent in diabetic women than current practice reflects [31,37]. Yet, women continue to experience treatment disparities.

#### 6.3.4. Antihypertensive Therapy and RAAS Blockade

Blood pressure control reduces cardiovascular events in women with diabetes, and RAAS blockade with ACE inhibitors or angiotensin receptor blockers appears to confer vascular endothelial benefits beyond blood pressure reduction, including attenuation of oxidative stress, restoration of bradykinin-mediated vasodilation, and reduction in renal and microvascular injury, which may be particularly relevant given the pronounced microvascular susceptibility of women with diabetes [8,38].

#### 6.3.5. Antioxidants

In diabetes, insulin resistance and hyperglycemia increase reactive oxygen species (ROS) through multiple pathways—including NADPH oxidase activation, mitochondrial electron transport chain overload, and advanced glycation end-product formation—overwhelming endogenous antioxidant defenses and elevating cardiovascular disease risk. Antioxidants, including vitamins C and E, selenium, glutathione, coenzyme Q10, carotenoids, and polyphenols, may help mitigate oxidative stress; however, their effects on clinically meaningful cardiovascular outcomes remain uncertain, optimal doses remain uncertain, and long-term clinical benefits have not been consistently demonstrated. Preclinical and limited clinical evidence suggests that *N*-acetylcysteine (NAC) and α-lipoic acid may improve endothelial function in diabetes, potentially by reducing oxidative stress, inflammation, and enhancing NO bioavailability. Nevertheless, antioxidant vitamin supplementation has not consistently reduced cardiovascular complications in individuals with diabetes [67,77,78]. Declining estrogen after menopause is also associated with increased oxidative stress, oxidized LDL, reduced NO bioavailability, endothelial dysfunction, and increased insulin resistance, potentially contributing to heightened cardiovascular risk in postmenopausal women [79]. Future research should account for sex and menopausal status when evaluating antioxidant therapies and supplementation, but these variables alone may not fully explain the heterogeneity observed in clinical trial outcomes [80].

#### 6.3.6. Hormonal Considerations

The role of hormone therapy (HT) in cardiovascular risk modification in perimenopausal and postmenopausal women with diabetes is complex and incompletely resolved [45]. Observational data from the general population suggest a cardiovascular benefit from timely initiation of HT in early menopause (the “timing hypothesis”), but the presence of diabetes significantly modifies this picture, as estrogen’s cardiovascular effects are fundamentally altered in the hyperglycemic environment [15,45]. A systematic review and meta-analysis of HT in women with T1D and T2D found benefits for glycemic control, but cardiovascular effects remained uncertain [72]. However, this mechanistic divergence presents a testable clinical hypothesis: the vascular response to HT in diabetic women likely depends on the extent of hyperglycemia-induced receptor reprogramming at the time of initiation. If the estrogen paradox is driven by ERα downregulation and eNOS uncoupling, then early initiation in perimenopausal women with strict glycemic control may preserve the physiological, cardioprotective estrogen signaling axis. Conversely, administering HT in the setting of chronic, uncontrolled hyperglycemia and established oxidative stress would likely redirect exogenous estrogen into maladaptive, superoxide-generating pathways, exacerbating endothelial injury. Current evidence does not support routine use of HT for primary cardiovascular prevention in women with diabetes, and its use should be individualized based on menopausal symptom burden, vascular risk profile, and glycemic control status [45,72]. In other words, hormonal interventions should be taken with strict caution.

## 7. Knowledge Gaps and Future Directions

The profound clinical and biological disparities identified in this review highlight the demand for substantial changes to the current cardiometabolic research agenda. To close the current gaps in female cardiovascular and preventative care, several topics should be addressed in future studies. First, cardiovascular outcome trials in diabetes should require sex-stratified primary and secondary analyses as a prerequisite for publication and regulatory evaluation, as the continued reliance on sex-aggregated outcomes obscures potentially important differences in treatment efficacy and safety [7]. Second, mechanistic studies are needed to clarify the interaction between estrogen receptor signaling and hyperglycemia across the full spectrum of diabetes types, disease duration, and hormonal life stages. Rigorous, reproducible studies are important for future diabetic care and can determine whether the estrogen paradox is reversible with improved glycemic control or whether it reflects longer-term receptor-level reprogramming [15,29]. Third, prospective longitudinal studies should characterize body composition trajectories, particularly visceral fat accumulation, lean mass changes, and ectopic fat deposition, as predictors of cardiovascular events in women with diabetes, essentially tracking the pathology of body composition [12,32]. This way, women’s metabolic risk assessment can move beyond the limitations of standard BMI stratification. Fourth, rather than generalizing exercise physiology from cohorts that are predominantly male, sex-stratified exercise intervention trials in both T1D and T2D are needed to generate the female-specific evidence base currently absent from exercise guidelines [34,73]. Specific exercise modalities can be evaluated and eventually recommended as more than general lifestyle advice, but rather as targeted interventions to preserve skeletal muscle. Fifth, novel sex-specific cardiovascular risk prediction models for diabetes, incorporating body composition, hormonal status, lifetime reproductive and pregnancy history and sex-specific biomarkers beyond traditional risk factors, are needed to replace the current sex-neutral tools that systematically underestimate risk in women [29].

## 8. Conclusions

Diabetes is a unique cardiovascular risk factor in women. The excess cardiovascular mortality observed in women with diabetes is not an inevitable consequence of the disease, but may be the result of a biological and clinical double-hit. Multiple mechanisms may play important roles: chronic hyperglycemia causes the normally protective effects of estrogen at the vascular endothelium to be reversed; the AGE-RAGE axis together with eNOS uncoupling leads to an oxidative and inflammatory environment that is specific to women; the different patterns of fat distribution according to sex worsen metabolic deterioration in a manner not reflected in BMI; and ongoing disparities in the way diabetes is managed in clinical practice add to the already high biological risk. Overcoming the first “hit” requires targeted interventions, such as resistance training and lipid-lowering therapy that specifically address visceral adiposity and endothelial dysfunction. Overcoming the second “hit” involves a conscious and ongoing commitment to sex-informed cardiovascular research, sex-stratified clinical evidence, and sex-aware clinical practice.

## Figures and Tables

**Table 1:** Potential mechanisms underlying the loss of female cardiovascular protection in women with diabetes.

Cardiovascular Risk Dimension	Healthy Physiological Baseline	Disruption from Diabetes & Hyperglycemia	Cardiovascular Pathophysiology & Risk	Targeted Clinical Interventions
Estrogen Receptor Signaling & eNOS Function	E2 binds ERα → activates PI3K/Akt → phosphorylates eNOS Promotes continuous NO production Supports vasodilation, anti-aggregation, and anti-adhesion	Oxidative stress downregulates ERα BH4 is oxidized to BH2 Causes eNOS uncoupling	Uncoupled eNOS produces superoxide instead of NO Promotes endothelial dysfunction and vascular damage FMD decreases in premenopausal women with T1D	Aerobic exercise/HIIT Restore eNOS and BH4 Reverse eNOS uncoupling BP control Individualized hormone therapy
Mitochondrial Homeostasis & AGE-RAGE Axis	Estrogen upregulates PGC-1α Supports mitochondrial biogenesis and respiratory capacity Enhances antioxidant defense	Glucose toxicity and ERα downregulation suppress PGC-1α AGEs accumulate AGE → RAGE activates NF-κB and NOX Reduces NO bioavailability	Impaired mitochondrial clearance Increased electron leakage and ROS generation Increased mitochondrial dysfunction Contributes to vascular damage	Combined aerobic and resistance training Reduce oxidative stress Limit AGE-RAGE accumulation Improve mitochondrial function
Inflammatory Cascades & Adhesion Molecules	Estrogen receptors suppress NF-κB signaling Upregulate IκBα Interact with p65 Limit inflammatory adhesion molecule transcription	Hyperglycemia and oxidative stress accelerate IκBα degradation Impaired estrogen signaling limits restoration of IκBα Disrupts normal NF-κB transrepression	Sustained NF-κB activation Chronic endothelial inflammation Increased hs-CRP, IL-6, and fibrinogen in diabetic women	Regular physical activity Reduce systemic inflammation Appropriate cardioprotective therapy, including statins and aspirin where indicated
Tissue Distribution & Lipotoxicity	Estrogen favors subcutaneous rather than visceral adipose storage Limits ectopic fat deposition in hepatic, visceral, and cardiorenal tissues	Subcutaneous storage capacity becomes overwhelmed Promotes visceral and ectopic fat accumulation Deposition occurs in pericardial, epicardial, hepatic, and skeletal muscle compartments	Visceral fat produces an atherogenic/inflammatory profile Epicardial fat may exert paracrine toxicity on myocardium Contributes to coronary microvascular dysfunction, myocardial steatosis, and HFpEF	Caloric restriction and exercise Target visceral adiposity GLP-1 receptor agonists Reduce body weight and lipotoxicity
Skeletal Muscle Mass & Insulin Sensitivity	Skeletal muscle is the primary site of insulin-stimulated glucose disposal Maintains systemic insulin sensitivity Contributes to resting energy expenditure	Reduced skeletal muscle mass May combine with obesity and insulin resistance Can contribute to sarcopenic obesity	Reduced glucose clearance Increased circulating FFAs Reduced cardiorespiratory fitness Reduced fitness → CV mortality risk	Combined aerobic + resistance training Preserve/build skeletal muscle Adequate dietary protein
Renin–Angiotensin–Aldosterone System (RAAS) Activity	Estrogen favors angiotensin-(1–7) and bradykinin-mediated vasodilation Suppresses vasoconstrictive AT1R signaling	Loss of estrogen receptor signaling disrupts RAAS balance Increased AT1R activation Increased angiotensin II and ACE activity	Sustained vasoconstriction EC inflammation Arteriolar remodeling Microvascular injury May → microvascular disease such as INOCA	Aggressive BP control ACE inhibitors or ARBs Reduce oxidative stress Protect microvascular function
Clinical Screening & Guideline Representation	Proactive CV risk screening Aggressive primary/secondary prevention Guidelines informed by adequate female representation in trials and female enrollment in clinical trials.	Women remain underrepresented in major CVOTs (~28–36%) May perpetuate sex-neutral guidelines Cardiovascular risk may be underestimated	Potential clinical “double-hit”: Increased biological risk Systematic undertreatment Lower likelihood of achieving LDL/BP goals Lower use of some cardioprotective therapies	Sex-stratified clinical-trial Aggressive lipid management Incorporate gestational diabetes, preeclampsia, and PCOS into risk models

→: indicates the direction of signaling or leads to downstream effects.

**Table 2:** Summary of Interventions to Restore or Preserve Female Cardiovascular Protection in Patients with Diabetes.

Intervention	Mechanism/Molecular Pathway	Evidence in Type1 Diabetes (T1D)	Evidence in Type2 Diabetes (T2D)	Female Perspective	Recommendation/ Considerations
Aerobic exercise (Brisk walking, Cycling, Swimming, Similar moderate-to-vigorous activity)	Endothelial function, NO signaling, insulin sensitivity, and mitochondrial function ↑ BP, dyslipidemia, and visceral adiposity ↓	HbA1c & insulin resistance ↓ VO_2_max, cardiorespiratory fitness & FMD ↑ Lipid profile & endothelial function improved [34,50].	Cardiometabolic risk ↓ Quality of life ↑ CV events unchanged [34].	Improved endothelial function in women with T2D and postmenopausal women; larger female-specific studies are needed [51,52].	Avoid >2 consecutive inactive days, target ≥150 min/week. T1D: monitor glucose; individualize carbohydrate/insulin reductions T2D: Combine with resistance training
High-intensity interval training (HIIT) (Short bursts of very intense exercise followed by longer periods of low- to moderate-intensity recovery)	Improves endothelial NO-dependent signaling and FMD	Improved FMD and VO_2_ peak versus moderate continuous training in young adults with T1D [53].	Controlled trials in T2D: Glycemic control, VO_2_max, vascular and cardiac function ↑ 39% lower liver fat with HIIT [54,55].	HIIT reduced abdominal fat and visceral fat post menopause. Glycemic control & cardiorespiratory fitness ↑; triglycerides, LDL cholesterol, and blood pressure ↓ [56,57].	Individualize optimal training intensity. Use continuous glucose monitoring in T1D due to glucose variability and hypoglycemia risk. More studies and longer-term studies are needed
Resistant Training (Exercises with free weights, weight machines, body weight, or elastic resistance bands)	Glucose uptake & cardiorespiratory fitness ↑ circulating FFAs ↓ [58,59].	Few T1D-specific studies directly evaluated cardiovascular benefits.	In adults with T2D: ↑ muscle strength (~50%) ↓ HbA1c (~0.57%) [34].	Muscle mass is inversely associated with HbA1c Low muscle mass → higher HbA1c Higher muscle mass → lower HbA1c [33].	Combining aerobics and resistance training may provide complementary benefits. Long-term randomized trials are needed
GLP-1 Receptor Agonists (Subcutaneous weekly, Oral daily administration)	Glycemic control and endothelial function ↑ Body weight, visceral adiposity, blood pressure, and vascular inflammation ↓ [49]	Observational study: Lower MACE & MI risk [60].	Major outcome trials of semaglutide, liraglutide, albiglutide, dulaglutide, and oral semaglutide: MACE ↓; Reduced cardiovascular death, MI, or stroke [49].	Sex-stratified analyses from large GLP-1 RA trials remain incompletely reported [7]	Require more T1D-focused studies Need more sex- and age-specific studies to understand the mechanism of action
SGLT2 inhibitors (Oral administration)	Natriuresis and mitochondrial energetics ↑ Blood pressure and cardiac preload/afterload ↓ [49].	Glycemic control & body weight improved; euglycemic DKA risk ↑; limited CV outcomes data [61].	HF hospitalization ↓; kidney & CV outcomes improved [49].	Sex-stratified analyses are inconsistent, limiting understanding of the female-specific perspective	Renal function should be monitored SGLT2 inhibitors are not approved in T1D for glycemic control due to the risk of DKA
Statins and Lipid-Lowering Therapy (Oral administration)	Cholesterol synthesis & inflammation ↓ LDL clearance & plaque stability ↑; Atherosclerotic Cardiovascular disease risk ↓ [62].	LDL-C & total cholesterol ↓; limited evidence for MACE or mortality reduction [63].	CARDS and HPS Trials reduced major CV/vascular events; ASPEN trial showed a nonsignificant reduction in CVD risk [62].	No dedicated female-specific randomized cardiovascular-outcome trials in diabetes [7].	Add statin therapy to lifestyle therapy; not a replacement for lifestyle management.
Antihypertensive Therapy and RAAS Blockade (Mostly Oral or Intravenous)	BP, oxidative stress & renal/microvascular injury ↓; Endothelial function ↑ [48].	Lowers blood pressure and vascular complications Slows progression toward diabetic nephropathy and CKD [64].	ADVANCE and FIDELIO-DKD trials showed reduced vascular, cardiovascular, and kidney outcomes in T2D [65].	Studies show women are frequently under-prescribed these therapies compared to men [66].	Consider age and sex before initiating therapy. Individualize treatment based on patient-specific factors.
Antioxidants (Mostly Oral or Intravenous)	Oxidative stress, lipid peroxidation & inflammation ↓, LDL oxidation resistance & vascular function ↑ [67].	Inconsistent outcome Improved endothelial function [68].	Glycemic, oxidative stress & vascular markers may improve; evidence remains heterogeneous and limited [67].	Vitamins C and E and beta-carotene did not prevent MACE. Women-focused trials should evaluate antioxidant therapies [69].	Large-scale trials are needed to determine cardiovascular benefit. Pharmacokinetics, bioavailability, and optimal dosing should be evaluated.
Hormone Therapy (Oral, Transdermal Patch)	May improve glycemia, insulin resistance, abdominal adiposity, inflammation, and endothelial function [70].	May relieve menopausal symptoms No evidence to improve blood glucose control or prevent cardiovascular disease [71].	Short-term postmenopausal hormone therapy improved HbA1c and fasting glucose, although study quality was variable [72].	Evidence directly concerns postmenopausal women, but cardiovascular outcome evidence remains limited.	Treatment should be individualized Should consider the timing of menopause CVD risk should be assessed before treatment

Abbreviations: ↑ indicates an increase; ↓ indicates a decrease; → indicates "leads to”.

## Data Availability

No new data were created or analyzed in this study. Data sharing is not applicable to this review article.
